# Antimicrobial resistance profile of *Staphylococcus aureus* isolated from patients, healthcare workers, and the environment in a tertiary hospital in Addis Ababa, Ethiopia

**DOI:** 10.1371/journal.pone.0308615

**Published:** 2024-08-15

**Authors:** Rajiha Abubeker Ibrahim, Shu-Hua Wang, Wondwossen A. Gebreyes, Jose R. Mediavilla, Gadissa Bedada Hundie, Zelalem Mekuria, Rozina Ambachew, Dejenie Shiferaw Teklu, Barry Kreiswirth, Degefu Beyene, Nega Berhe

**Affiliations:** 1 Akililu Lemma Institute of Pathobiology, Addis Ababa University, Addis Ababa, Ethiopia; 2 Ethiopian Public Health Institute, Addis Ababa, Ethiopia; 3 Ohio State Global One Health (GOH) LLC, Addis Ababa, Ethiopia; 4 Internal Medicine Department, Infectious Disease Division, College of Medicine, The Ohio State University, Columbus, Ohio, United States of America; 5 The Ohio State University, Global One Health initiative (GOHi), Columbus, Ohio, United States of America; 6 Colleges of Veterinary Medicine, The Ohio State University, Columbus, Ohio, United States of America; 7 Center for Discovery and Innovation, Hackensack Meridian Health, Nutley, New Jersey, United States of America; 8 Department of Microbiology, Immunology and Parasitology, St. Paul’s Hospitals Millennium Medical College (SPHMMC), Addis Ababa, Ethiopia; Tribhuvan University, NEPAL

## Abstract

*Staphylococcus aureus* infection and colonization in patients may be transmitted to healthcare providers and the environment and subsequently cause healthcare-associated infections in other patients. Pathogenic *S*. *aureus* strains produce virulence factors, such as Panton-Valentine Leukocidin (PVL), that contribute to the severity of infections and aid in their spread. The emergence of antimicrobial resistance (AMR) is additional concern with respect to *S*. *aureus* infection. In this study, the virulence genes and antibiotic resistance profiles of *S*. *aureus* were characterized from patients’ clinical isolates, healthcare workers’ (HCWs’) nasal colonization screenings, and the environment at a tertiary healthcare hospital in Addis Ababa, Ethiopia. A total of 365 samples were collected from September 2021 to September 2022: 73 patients’ clinical specimens, 202 colonization screenings from HCWs, and 90 hospital environment’s swabs. Fifty-one (25.2%) HCW and 10/90 (11.1%) environment *S*. *aureus* isolates were identified. Among the 134 isolates, 10 (7.5%) were methicillin-resistant *S*. *aureus* (MRSA). Three (4.1%), five (9.8%), and two (20.0%) of the MRSA isolates were identified from the patients, HCWs, and the environment, respectively. Overall, 118 (88.1%) were ampicillin and penicillin resistant; 70 (52.2%) were trimethoprim sulfamethoxazole resistant; and 28 (20.9%) were erythromycin resistant. *S*. *aureus* isolates from patients were more resistant to antibiotics than isolates from HCWs or the hospital environment (p<0.05). A total of 92/134 (68.6%) isolates possessed the *lukf*F-PV gene, which was identified in 62 (85.0%), 26 (51.0%), and 4 (40.0%) of the patient, HCWs, and the environment, respectively. The proportion of *lukf*F-PV gene containing *S*. *aureus* isolated from patient samples was statistically significant. Four (40.0%) of the MRSA isolates also had the *lukf*F-PV gene. The identification of highly AMR and virulence factors from patients, HCWs and the environment is concerning. Further studies are needed to identify potential transmission links and improve infection prevention and control.

## Introduction

*Staphylococcus aureus* is a widespread organism that is also found as a normal flora of human body. Approximately 20–30% of the human populations are reported to be colonized with persistent nasal carriage of *S aureus*, while 60% are intermittent carriers, and another 20% are non-carriers [1, 2]. *S*. *aureus* colonization of patients has been connected to subsequent infection [3], and healthcare workers (HCWs) have been identified as potential reservoirs and possible source for the cross-transmission of pathogens associated with healthcare-associated infections (HAIs) [4, 5].

*S*. *aureus* infections can affect any part of the body from minor skin infections to serious, life-threatening pneumonia and bacteremia [6, 7]. The production of virulence factors by pathogenic strains, such as expression of a cell-surface protein that binds and inactivates antibodies, as well as the production of powerful protein toxins like staphylococcal enterotoxin and Panton-Valentine Leukocidin (PVL), can aid in the spread of infections [8] PVL is a pore-forming toxin largely responsible for skin and soft tissue infection. There is a significant variation in the prevalence of PVL among methicillin-susceptible *S*. *aureus* (MSSA) and methicillin-resistant *S*. *aureus* (MRSA) infections worldwide. The prevalence of PVL toxin has been reported to be increasing in Africa [9, 10].

Emergence of antimicrobial resistance (AMR) in healthcare facility is a growing concern due to increasing prevalence of multidrug resistant organisms (MDROs) and limited availability of effective antimicrobial therapeutics for treatment. In Ethiopia, the majority of studies on *S*. *aureus* AMR patterns rely on culture-based pathogen identification and drug susceptibility testing. However, there is a limited understanding of the molecular resistance and virulence gene profiles of *S*. *aureus* in Ethiopia [14]. This study aimed to characterize the antibiotic resistance and virulence gene profiles of *S*. *aureus* isolates obtained from patient clinical samples, healthcare worker colonization screenings, and environmental sampling at a tertiary healthcare hospital in Addis Ababa, Ethiopia.

## Material and methods

### Study design, sampling procedures and data collection

This cross-sectional study was conducted at St Paul’s Hospital Millennium Medical College (SPHMMC) in Addis Ababa, Ethiopia. Patients’ clinical specimen, healthcare workers’ nasal swab and hospital environment swabs were the source of samples utilized in this study.

**Patients:** All clinical specimens submitted for routine culture that was positive for *S*. *aureus* at the SPHMCC from September 2021 to September 2022 were included in the study. The isolates were kept at -80 freezer using 20% glycerol and tryptic soya broth in a cryo-tube for further testing. Patient demographics, diagnosis, specimen type and hospital ward location at the time of specimen submission were collected from hospital medical records. The medical record of the patients was accessed from October 3–7, 2022 and isolates were subculture for testing at October 10, 2022.

**Healthcare workers (HCWs):** Nasal swabs were collected from volunteer HCWs (doctors, nurses, laboratory personnel, and environmental cleaners) from September 12, 2021 to August 30, 2022. HCWs were recruited from target hospital units (Emergency department (ED), gynecological (GYN), medical ward (MED), pediatric ward (PEDs), surgical unit (SURG), and laboratory (LAB). HCW sample size was calculated based on a prior colonization study prevalence [15]. Using single population formula, a sample size of 170 was estimated, plus an additional 20% of the sample size, for a total of 202 HCWs. Consent was obtained prior to specimen collection. The HCWs, socio-demographic information including age, sex, years of work experience, and hospital unit location was collected using a short questionnaire. Nasal swab samples were collected from the HCW. The swab was inserted approximately 2 cm into one nostril and rotated against the anterior nasal mucosa for 3 seconds. The same swab was inserted into the other nostril and rotated for 3 seconds. Upon completion, the swab was placed into the Amies transport media with charcoal. The samples were transported to SPHMMC microbiology laboratory. The samples were cultured in mannitol salt agar plate.

**Hospital environmental sampling:** Environmental swab samples were collected from predetermined the hospital’s environment high touch or high contact surfaces and classified into three categories: Patient areas, staff areas (limited to hospital staff), and general public areas. Environmental swab specimens collected from patient areas included patient rooms (intravenous [IV] pole, bed railing, bedside table, and shelf), procedure rooms (table, medical chart, medical chart shelf), and medical supplies (oxygen, mechanical ventilator). Samples collected in staff area included nurse station (medication tray, table), documentation (file tray, medical chart), office supplies (Table, chair and computer), laboratory (chair and bench) and medical supplies and equipment (medication shelf, ultrasound). Samples were also collected from public areas: reception (shelf, hand wash sink); waiting area (chair handle and chair); corridor (elevator buttons and stair railings). Daily cleaning of the environment takes place in the morning, thus all 90 of the environmental samples were all collected after 2:00 pm. Samples were collected using two pre-moisten with sterile saline water swabs and Amies transport media were used to transport the sample to the laboratory [16].

### Identification of *S*. *aureus*

Preliminary *S*. *aureus* identification was performed at SPHMMC microbiology laboratory using pre-enrichment, non-selective and selective media as previously described [16]. In summary, swabs from HCWs and the environment were placed in 2 mL of tryptone soy broth with NaCl and incubated aerobically at 35°C for 24 hours, after which 50-μL aliquots of the broth were inoculated on to mannitol salt agar plates. Colonies were selected based on colony morphology and were then sub-cultured on to tryptic soy agar (TSA). Positive catalase and coagulase reactions were considered presumptive positive for *S*. *aureus*.

### Confirmation of MSSA, MRSA and antimicrobial susceptibility

Isolate confirmation and antimicrobial susceptibility testing (AST) were performed for all presumptive isolates at EPHI Clinical Bacteriology and Mycology Laboratory using BD Phoenix M50 system (BD Diagnostic Systems, Franklin Lakes, NJ, USA) version 6.8.1 PMIC/ID-111 panel following manufacturer’s procedures [17]. In addition, isolates were determined as either MSSA or MRSA based on the resistance to cefoxitin as determined by minimum inhibitory concentration (MIC> = 8). The isolates were stored at -80°C in tryptic soy broth (TSB) containing 20% glycerol for further molecular testing.

### Nucleic acid isolation and amplification of 16S rRNA, *mecA*, *lukF-PV* and *spa* genes

Genomic DNA was extracted using a commercially available QIAamp DNA Mini kit (QIAGEN, Hilden, Germany), following the manufacturer’s protocol [18]. DNA extraction and polymerase chain reaction (PCR) tests were performed at EPHI. The extract was stored at -20°C until further analysis. For PCR amplification and analysis of 16S rRNA, *mecA*, *lukF-PV* and *spa* genes, the QIAGEN Microbial DNA qPCR Multi-Assay Kit (QIAGEN, Hilden, Germany) was used according to the manufacturer’s procedure [19].

### Statistical analysis

STATA version 16.2 (Stata Corp LLC, College Station, TX, USA) was used for data analysis. Descriptive statistics were generated to summarize cefoxitin screening results and *S*. *aureus* prevalence by sampling site. Logistic regression models were fitted to calculated odds ratio and determine the association between antibiotic resistance and sample source; *lukF-PV* carriage and antimicrobial resistance; MRSA status and independent factors such as age, gender, departments and HCW’s years of work experience.

### Ethical clearance

The study protocol was approved by the EPHI Institutional Review Board (IRB, EPHI-IRB-029-2017) and SPHHMC IRB (PM23/352). Written consent was obtained from healthcare providers for colonization screening. Retrospective patient data and archived isolates were used; Anonymous analysis was conducted; authors did not have access to information that could identify individual participants, and the ethics committee waived patient consent.

## Results

### Socio-demographic characteristics of study participants

#### Patients

Out of 73 patients with *S*. *aureus* positive culture, 44 (60.3%) were female. The female patients had a mean age of 24.1 years and a median age of 26 years. In contrast, the male patients had a mean age of 16.7 years and a median age of 7 years. For females, the age group of 26–39 made up the majority of participants, whereas for males, it was one year and younger (Table 1).

**Table 1:** Distribution of patient (N = 73) age-group, gender, and location in St. Paul’s Hospital Millennium Medical College (SPHMMC) in Addis Ababa, Ethiopia.

	Age n (%)	Sex n (%)		Departments n (%)					
Years		Female	Male	ED	GYN	MED	OPD	PED	SURG
**< = 1**	20 (27.4)	8 (18.2)	12 (41.4)	0	NA	1 (8.3)	1 (10.0)	18 (72.0)	0
**2–14**	8 (10.9)	1 (2.3)	7 (24.2)	0	NA	1 (8.3)	0	7 (28.0)	0
**15–25**	16 (21.9)	13 (29.6)	3 (10.3)	4 (44.4)	1 (11.1)	3 (30.0)	4 (44.4)	NA	4 (36.3)
**26–39**	20 (27.4)	17 (38.6)	3 (10.3)	3 (33.3)	7 (77.8)	3 (30.0)	3 (44.4)	NA	4 (36.3)
≥ 40	9 (12.3)	5 (11.4)	4 (13.8)	2 (22.2)	1 (11.1)	2 (20.0)	1 (11.1)	NA	3 (27.3)
**Total**	**73 (100)**	**44 (60.3)**	**29 (34.2)**	**9 (12.3)**	**9(12.3)**	**10 (13.7)**	**9 (12.3)**	**25 (34.3)**	**11 (15.7)**

Abbreviations: ED, Emergency Department; GYN, Gynecology ward; MED, Medicine ward; OPD, outpatient unit; PED, Pediatric ward; SURG, Surgical ward; NA, not applicable.

**Notes**: 95% Confidence Interval were used.

### Health care workers

Out of the 202 HCWs who enrolled and consented to participate in the study, 103 (51.0%) were males. Although the age of HCW range from 21 to 49 years, the majority (65.8%) of them were in the 21–29 age group, with a mean age of 29 and a median age of 28 (Table 2).

**Table 2:** Distribution of health care workers age-group (N = 202), gender, and location in St. Paul’s Hospital Millennium Medical College (SPHMMC) in Addis Ababa, Ethiopia.

Sex		Department	
**Female**	99 (49.0)	**Emergency**	21 (10.40)
**Male**	103 (51.0)	**Gynecology**	13 (6.44)
**Age group**		**Laboratory**	18 (8.91)
**21–29**	133 (65.8)	**Medicine**	53 (26.24)
**30–39**	57 (28.2)	**Pediatric**	42 (20.79)
**40–49**	12 (6.0)	**Surgical**	55 (27.23)

### Identification of MSSA and MRSA from patients, HCWs and environment

**Fig 1:**
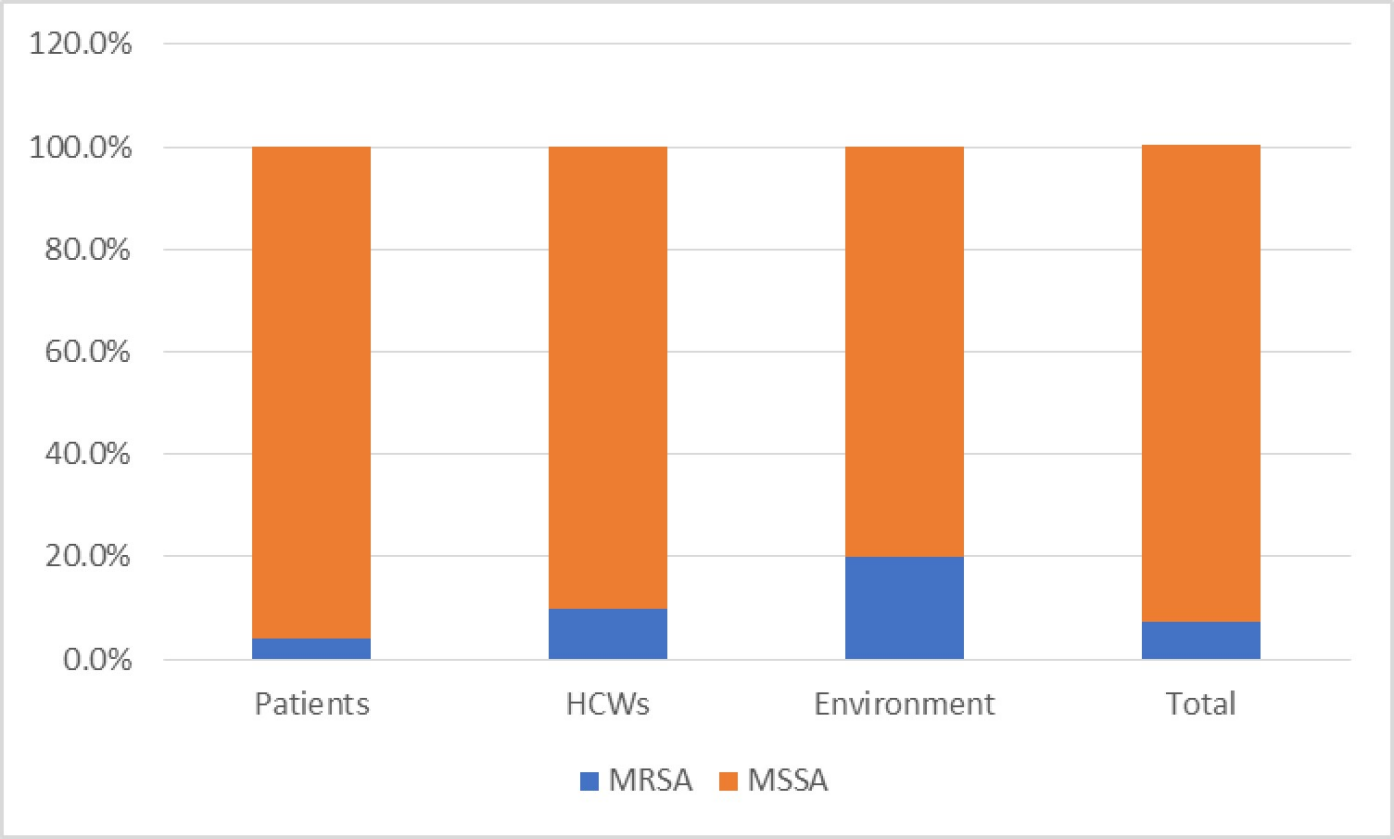
Methicillin-resistant and methicillin-sensitive Staphylococcus aureus by sample source. Abbreviations: MRSA: methicillin resistant; MSSA: methicillin susceptible resistant; HCW: Healthcare worker.

Overall, a total of 124 MSSA and 10 MRSA isolates were identified. Out of the 124 MSSA, 70 isolates were from patients’ clinical specimen, 46 from HCW nasal colonization, and 8 from hospital environmental source (Fig 1). Of the 10 MRSA isolates, 3 were from patient’s clinical specimen, 5 from HCW nasal colonization, and 2 from hospital environmental source. There was no significant difference in MRSA prevalence among different sample sources.

Of the total of 73 patient isolates, 3 (4.1%) were MRSA. The distribution of *S*. *aureus* isolation from different departments varies with higher percentage in the pediatric ward and the lowest in the outpatient department (Table 1).

Among the total of 202 HCW participants, 51 (25.2%) were colonized with S. *aureus* of which 5 (9.8%) were found to be MRSA. The distribution of *S*. *aureus* varied between different age groups. Higher frequencies of *S*. *aureus* and MRSA were found in the age group 21–29 years, but the difference was not statistically significant. The percentage of *S*. *aureus* and MRSA among HCWs was relatively higher in the surgery department, 11 (21.6%) and 2 (40.0%), respectively; however, this difference was not statistically significant. Similarly, there were no significant associations between isolation of *S*. *aureus* and years of work experience among HCWs (S1 Table).

Out of the 90 environmental samples, 10 (11.1%) yielded *S*. *aureus* isolates. Two of these isolates (20.0%) were MRSA. One MRSA isolate was recovered from the patient area, while the other was from the staff area.

### Antimicrobial resistance

Antimicrobial susceptibility testing was performed on 134 *S*. *aureus* isolates: 73 from patients, 51 from HCWs, and 10 from environment (Table 3). One hundred eighteen 118 (88.1%) were resistant to ampicillin and penicillin; 70 (52.2%) were resistant to trimethoprim sulfamethoxazole (SXT); 28 (20.9%) were resistant to erythromycin; 22 (16.4%) were resistant to tetracycline; 21 (15.7%) were resistant to clindamycin; 5 (3.7%) were resistant to ciprofloxacin; and 2 (1.5%) were resistant to ceftaroline. A total of 11 (8.2%) isolates were resistant to oxacillin, but one isolate was negative for methicillin resistance using the cefoxitin test. A total of 10 (7.4%) isolates were therefore, identified as MRSA (i.e., the resistance was resulting from the presence of *mecA* gene) (Table 3). None of the isolates were resistant to vancomycin, linezolid, daptomycin, nitrofurantoin and high-level mupirocin.

**Table 3:** Antimicrobial resistance patterns of methicillin-resistant and methicillin sensitive *Staphylococcus aureus*.

Antibiotics	Total (N = 134)	Total MSSA (N = 124)	Total MRSA (N = 10)
	Resistance n (%)	Resistance n (%)	Resistance n (%)
**Ampicillin**	118 (88.1)	108 (87.1)	10 (100.0)
**Cefotaxime**	8 (5.9)	0 (0.0)	10 (1000.0)
**Cefoxitin**	10 (7.4)	0 (0.0)	10 (100.0)
**Ceftaroline**	2 (1.5) *	0 (0.0)	2 (20.0) *
**Ciprofloxacin**	5 (3.7)	0 (0.0)	5 (50.0)
**Clindamycin**	21 (15.7) *	18 (14.5)	3 (30.0)
**Daptomycin**	0 (0.0)	0(0.0)	0 (0.0)
**Erythromycin**	28 (20.9)	22 (17.7)	6 (60.0)
**ICR**	15(11.2)	14(11.3)	1 (10.0)
**Gentamicin**	2 (1.5)	0 (0.00)	2 (20.0)
**Levofloxacin**	6 (4.5) *	2 (1.7)	4 (40.0) *
**Linezolid**	0 (0.0)	0 (0.0)	0 (0.0)
**Moxifloxacin**	4 (3.0) *	0 (0.0)	4 (40.0)
**Mupirocin HL**	0 (0.0)	0 (0.0)	0 (0.00)
**Nitrofurantoin**	0 (0.0)	0 (0.0)	0 (0.0)
**Oxacillin**	11 (8.2)	1 (0.8)	10 (100.0)
**Penicillin G**	118 (88.1)	108 (87.1)	10 (100.0)
**Rifampicin**	1 (0.75)	0 (0.0)	1 (10.0)
**SXT **	70 (52.2)	63 (50.8)	7 (70.0)
**Teicoplanin**	4 (3.0)	4 (3.2)	0 (0.0)
**Tetracycline**	22 (16.4) *	8 (15.3) *	3 (30.0)
**Tigecycline**	10 (7.5) *	9 (7.3) *	1 (10.0)
**Vancomycin**	0 (0.0)	0 (0.0)	0 (0.0)

**Abbreviations**: MSSA, methicillin-sensitive *Staphylococcus aureus*; MRSA, methicillin-resistant *Staphylococcus aureus*; HCW, healthcare worker; Environ., environmental; ICR, inducible clindamycin resistance HL, high level mupirocin resistance; SXT: Trimethoprim-Sulfamethoxazole; *Intermediate resistance was classified as resistant.

All of the MRSA isolates were resistant to cefotaxime, whereas 7 (70.0%) of the MRSA isolates were also resistant to SXT; 6 (60.0%) were resistant to erythromycin; 5 (50.0%) were resistant to ciprofloxacin; 4 (40.0%) were resistant to levofloxacin; 3 (30.0%) were resistant to tetracycline; 2 (20.0) were intermediate resistant to ceftaroline; and 1 (10%) was resistant to rifampicin (Table 3).

Among the 124 (92.6%) MSSA isolates 108 (87.1%) were resistance to benzylpenicillin; 63 (50.8%) were resistant to SXT and 22 (17.7) were resistant to erythromycin. In addition, 18 (14.5%) and 19 (15.3%) isolates were resistance to clindamycin and tetracycline, respectively.

### Patients

Of the total 73 *S*. *aureus* isolates from patients tested for AST, 67 (91.8%) were resistant to ampicillin, and 66 (90.4%) were resistant to penicillin; 46 (63.0%) were resistance to SXT; 12 (16.4%) were resistant to tetracycline; 13 (17.8%) were resistant to erythromycin; 8 (11.0%) were resistant to clindamycin; 4 (5.5%) were resistant to teicoplanin; and 1 (1.4%) was resistant to ceftaroline (Table 4).

**Table 4:** Antimicrobial resistance patterns of *Staphylococcus aureus* strains from patients, Health Care Workers, and hospital environment.

Antibiotics	Patient (N = 73) Resistance n (%)	HCW (N = 51) Resistance n (%)	Environment (N = 10) Resistance n (%)
**Ampicillin**	67 (91.8)	41 (80.4)	10 (100.0)
**Cefotaxime**	3 (4.1)	5 (9.8)	2 (20.0)
**Cefoxitin**	3 (4.1)	5 (9.8)	2 (20.0)
**Ceftaroline**	1 (1.4) *	1 (2.0) *	0 (0.0)
**Ciprofloxacin**	1 (1.4)	2 (3.9)	2 (20.0)
**Clindamycin**	8 (11.0)	10 (19.6)	3 (30.0) *
**Daptomycin**	0 (0.0)	0 (0.0)	0 (0.0)
**Erythromycin**	13 (17.8)	12 (23.5)	3 (30.0)
**ICR**	7(9.6)	8(15.6)	0(0.0)
**Gentamicin**	0 (0.00)	1 (2.0)	1 (10.0)
**Levofloxacin**	1 (1.4)	2 (3.9) *	3 (30.0)
**Linezolid**	0 (0.0)	0 (0.0)	0 (0.0)
**Moxifloxacin**	1 (1.4)	1 (2.0)	2 (20.0)
**Mupirocin HL**	0 (0.0)	0 (0.0)	0 (0.0)
**Nitrofurantoin**	0 (0.0)	0 (0.0)	0 (0.0)
**Oxacillin**	3 (4.1)	6 (11.8)	2 (20.0)
**Penicillin G**	66 (90.4)	42 (82.4)	10 (100.0)
**Rifampicin**	0 (0.0)	0 (0.0)	1 (10.0)
**SXT **	46 (63.0) `	22 (43.1)	2 (20.0)
**Teicoplanin**	4 (5.48)	0 (0.0)	0 (0.0)
**Tetracycline**	12 (16.4)	10 (19.6) *	0 (0.0)
**Tigecycline**	7 (9.6)	3 (5.9) *	0 (0.0)
**Vancomycin**	0 (0.0)	0 (0.0)	0 (0.0)

SXT, trimethoprim-sulfamethoxazole

### Health care worker

Of the total 51 HCW *S*. *aureus* isolates tested for AST, 41 (80.4%) and 42 (82.4%) were resistant to ampicillin and penicillin respectively; 22 (43.1%) were resistance to SXT; 12 (23.5%) were resistant to erythromycin; 10 (19.6%) resistant to tetracycline; 10 (19.6%) were resistant to clindamycin; 1 (2.0%) was resistant to ceftaroline. Six (11.7%) isolates were resistant to oxacillin, but one was cefoxitin-screening negative. Five (9.8%) isolates were therefore identified as MRSA (Table 4).

### Environment

All of the10 *S*. *aureus* strain isolated from hospital environment were resistant to both ampicillin and penicillin while 3 (30.0%) were resistant to clindamycin, erythromycin and levofloxacin. None of the environmental isolates were resistant to ceftaroline and tetracycline (Table 4).

### Associations between antibiotic resistance and sample sources

Table 5 below shows the association between antimicrobial resistance and sample source (patients vs. healthcare workers in reference to environmental sample) for several different antibiotics. The table includes odds ratios (OR) and p-values for each antibiotic. Significantly high level of resistance was observed in levofloxacin and ciprofloxacin in environmental samples compared to patient’s sample. While the odds of *S*. *aureus* to be resistant to SXT among patients are 6.81times more, that is statically significant.

**Table 5:** Association of antimicrobial resistance and sample source.

Antibiotics	Patients		HCW	
	OR	p-value	OR	p-value
**Ampicillin**	2.724	0.070	-	-
**Cefoxitin**	0.170	0.074	0.430	0.360
**Cefotaxime**	0.170	0.074	0.435	0.365
**Ceftaroline**	-	-	0.694	0.798
**Ciprofloxacin**	0.056	**0.024**	0.205	0.140
**Clindamycin**	0.287	0.112	0.569	0.467
**Daptomycin**	-	-	-	-
**Erythromycin**	0.506	0.366	0.717	0.665
**Gentamicin**	-	-	0.180	0.240
**Levofloxacin**	**0.032**	**0.005**	0.095	**0.019**
**Oxacillin**	0.171	0.074	0.533	0.486
**Penicillin G**	2.020	0.194	NA	NA
**Tetracycline**	0.806	0.650	NA	NA
**Tigecycline**	1.697	0.460	NA	NA
**SXT**	6.815	**0.020**	3.034	0.186

**Notes:** 95% confidence intervals were used

**Abbreviations**: HCW, Health care worker; OR, odds ratio; SXT, trimethoprim-sulfamethoxazole

### *spa*, *mecA*, and *lukF-*PV gene detection

All 134 isolates tested positive for the presence of the *spa* genes, whereas the *mec*A gene was only detected in 10 (7.4%) MRSA isolates. The *lukF-PV* gene was detected in 92/134 (68.6%) of the *S*. *aureus* isolates, including in 62 (85.0%), 26 (51.0%) and 4 (40.0%) of the patients, healthcare worker, and environmental isolates, respectively.

Four (40%) of the 10 *mecA* positive MRSA isolates possessed the *lukF-PV* gene, compared to 87/124 (70.2%) of MSSA isolates. Among the latter, 60/87 (69.0%) were isolated from patients, while 25/87 (28.7%) were isolated from health care workers and 2 (2.3%) from environmental samples. Significant associations were observed between isolates from patients and the presence of the *lukF-PV* gene in reference to environmental samples (Table 6). *lukF-PV* gene positive isolates had a 4.54 times higher probability of being SXT-resistant; while oxacillin-resistant isolates were less common among isolates that tested positive for *lukF-PV* gene (Table 6). However, no associations were found between *lukF-PV* prevalence and departmental location.

**Table 6:** Association of resistant isolate and, *lukF-PV* prevalence, and specimen source, *lukF-PV* prevalence and isolate department, MRSA and specimen source.

All samples antimicrobial resistance and PVL association			
**Antibiotics**	**PVL**	**OR**	**p-value**
**Cefoxitin**	4(40.0)	0.280	0.062
**Ampicillin**	80(67.8)	0.950	0.940
**Cefotaxime**	4(40.0)	0.280	0.062
**Ceftaroline**	1(50.0)	0.470	0.593
**Ciprofloxacin**	2(40)	0.278	0.171
**Clindamycin**	13(61.9)	0.729	0.522
**Erythromycin**	15(53.6)	0.456	0.071
**Levofloxacin**	3(50.0)	0.454	0.347
**Oxacillin**	4(36.4)	**0.236**	**0.028**
**Penicillin G**	79(66.9)	0.675	0.519
**Tetracycline**	16(72.7)	1.316	0.597
**Tigecycline**	8(80.0)	1.975	0.402
**SXT**	58(82.8)	**4.540**	**<0.001**
**Association of PVL and sample source**			
**Sample source**	**PVL**	**OR**	**p-value**
**Patients**	62(84.9)	13.150	**0.001**
**HCWs**	26(50.9)	2.420	1.190
**Environment**	1	1	1

**Notes:** 95% confidence intervals were used.

**Abbreviations**: HCW, Health care worker; OR, odds ratio; significant *p*-value (< = 0.05) are shown in boldface

## Discussion

The emergence and persistent spread of drug-resistant *S*. *aureus* have become one of the most frightening problems facing the world today. In the present study, we found that the majority of isolates were MSSA; nevertheless, these were resistant to commonly prescribed oral antibiotics such as penicillin, SXT, tetracycline, erythromycin and clindamycin resulting in limited treatment options for outpatients. In addition, over two thirds of the isolates harbored the *lukF-*PV gene encoding PVL, indicating increase virulence in the isolates.

Association between *S*. *aureus* / MRSA infection and host attributes such as age and gender have been searched for in the present study. The most frequent age ranges for female patients were 26 to 39 years old and for male it was under 1 year of age. With the exception of four blood samples, most of the children under a year old were from outpatient and had pus or abscesses. Since the children were not hospitalized and the majority of their *S*. *aureus* infections were community associated, an outbreak in the hospital was not likely the source of the children’s infection.

Male children displayed a slightly higher prevalence of *S*. *aureus* infection among other age groups. Overall, there was a slightly higher percent of female patients in our study, in contrast to other studies that have reported a higher prevalence of *S*. *aureus* infection in males [7]. Majority of female patients were in the reproductive age and some of them were breast feeding (data not shown). *S*. *aureus* transmission from mother to child has been reported in different studies [20, 21]. Given the high infection rate among young mothers and infants found in the present study, further studies are needed in our patient population to conclude the risk of mother-to-child transmission of *S*. *aureus*.

*S*. *aureus* exhibit many virulence factors that are important or necessary for its survival and ability to infect humans. One of the key components of *S*. *aureus* pathogenicity that helps it avoid human immunological reactions is staphylococcal protein A, [22]. In this study, the spa gene was found in every isolate; however, in other studies, the difference in primer binding sites caused up to 3% of the isolates not to detect the gene [23]. Panton-Valentine Leukocidin (PVL) is another virulence gene expressed by *S*. *aureus* and is known to be associated with severe form of infection. It is usually associated with skin and soft tissue infection and sepsis [24, 25]. In this study over two-third of the isolates possessed PVL gene and this is in agreement with previous study conducted in Ethiopia indicating that there is a high prevalence virulent *S*. *aureus* strains encoding *pvl* gene in the county [26].

*S*. *aureus* may carry the *mecA* gene, which confers resistance to methicillin, penicillin, and other drugs that resemble penicillin. In this study the detection of MRSA in patients was very low compared to other studies conducted in Ethiopia [12, 13]. Other studies utilizing molecular techniques also revealed very low frequencies of MRSA cases in Ethiopia [14, 27]. This may be because the majority of the study conducted used Kirby-Bauer disc diffusion method by cefoxitin screening or oxacillin test (which needs E- test for confirmation and most of the laboratories do not have E tests). Similar condition was reported in other East Africa country [28].

Interestingly, the age range of the majority of HCWs was between 21 and 29 years of age, indicating a young workforce. This is consistent with the report that showed the majority of healthcare professionals in Ethiopia were under 35 years old [29]. This could be the cause of the lack of significant association between MRSA carriage and age group. The percentage of *S*. *aureus* identification was comparable to other studies in Ethiopia [30, 31]. The HCWs’ department and year of experience had no significant association with the presence of MRSA. In contrast, other studies investigating the nasal carriage of *S*. *aureus* and MRSA, reported significant associations with one or more of the aforementioned factors [31, 32]. Similarly, there was no statistically significant association between the hospital ward and the sample collection area among environmental isolates of MRSA.

In environmental samples, the frequency of MRSA was slightly higher. In addition, fluoroquinolones have shown significantly high level of resistance in environmental samples compared to other samples sources. This may be because the bacteria in a hospital environment are more likely to develop antibiotic resistance due to high use [33, 34]. A study conducted elsewhere reported high MRSA contamination in hospital surfaces during no outbreak periods [35]. A study conducted in another hospital in Addis Ababa reported a comparable MRSA colonization rate of 9.8% among health care workers [31].

In this investigation, all *S*. *aureus* isolates were susceptible to antibiotics such as daptomycin, linezolid, nitrofurantoin, and vancomycin. These findings were in line with those of other East African studies [14, 36, 37]. For instance, linezolid is only authorized for use in certain centers and is only designated for the treatment of multidrug-resistant tuberculosis [38]. Other antibiotics, such as daptomycin, are not included in the national drug list [38].

Since every isolate was responsive to the previously listed antibiotics, daptomycin and vancomycin are prescribed for serious infections such as bacteremia/ osteomyelitis caused by MRSA or for those with penicillin allergy who have MSSA and are unable to take oxacillin. It ensures that patients with severe invasive *S*. *aureus* infections may still have recourse to potent antibiotics. By contrast, a substantial percentage of regularly used antibiotics, including oral medications such as penicillin, SXT, and tetracycline, demonstrated high levels of resistance. Studies carried out elsewhere in Ethiopia have revealed a similar resistance profile [11, 14]. This is likely connected to self-medication and overuse of antibiotics in community settings [37, 39]. There are also reports indicating that many antibiotics are prescribed empirically and unnecessarily [40, 41].

An isolate from HCW sample displayed resistance to oxacillin but was susceptible to cefoxitin. Cefoxitin is more reliable indicator of *mecA* gene carriage, and molecular analysis confirmed that this isolate did not harbor the *mecA* gene. This finding highlights that resistance to oxacillin is mediated by resistance mechanisms other than *mecA* [42].

Despite the fact that ceftaroline has been shown to have increased activity against MRSA in many countries globally [14, 43, 44], it is concerning that we have identified ceftaroline resistant isolates. Especially because this antibiotic is not included in Ethiopia’s list of essential medications and is not available at SPHMMC [38]. Ceftaroline may be available in some hospitals in Addis Ababa and this resistance could be derived from selection pressure. It is also alarming that of the two MRSA isolates with intermediate resistance to ceftaroline, one originated from a patient and the other from a healthcare provider.

The virulence gene *lukF-PV* was present in around two-thirds of the isolates, including 40% of the MRSA isolates. When compared isolates from other sources, patient sample isolates displayed greater levels of resistance to SXT which demonstrated statistically significant associations. The detection of the virulence gene *(lukF-PV*) was relatively higher across all sample sources, with patient samples displaying the most significant statistical correlation. This may be linked to the possibility that isolates that produce infection are more virulent as compared to strains identified in environment. A study from East Africa suggested that the *pvl* toxin may be increasing in prevalence among clinical isolates [45].

Whereas a previous study in Ethiopia did not find the *lukF-PV* gene in MRSA isolates, this study observed the *lukF-PV* gene in *mecA* positive isolates [46]. Similar reports from other East African countries suggest that MRSA strains harboring PVL represent significant pathogenic strains in patients with skin and soft tissue infections [47].

## Conclusion

Although, some of the antibiotics such as vancomycin, linezolid and daptomycin were complete susceptible to *S*. *aureus*, there is higher resistance against common antibiotics such as penicillin, trimethoprim sulfamethoxazole and erythromycin and clindamycin. Ceftaroline resistance is concerning as well, and since testing for resistance is not regularly done, additional study is required to determine the true prevalence of the resistance. The prevalence of MRSA found in patients, healthcare workers, and the environment was comparatively low. The detection of MSSA or MRSA was shown to have no association with sample source, sample collection department, gender and age. Moreover, patient isolates had significant association with high prevalence of *lukF-PV* gene. These findings demonstrate the emergence of antimicrobial resistance to new drugs and the prevalence of virulent *S*. *aureus* strains in the study site. Further studies to identify potential transmission links and improved infection prevention and control and environmental cleaning are needed to prevent spread. In addition, we recommend enforcing proper use and regulation of antibiotics in Ethiopia.

## Supporting information

S1 Table Demographics of health care workers and positive isolates from each sample and correlation of the age, sex, department, work experience, profession and level of education to isolation of *S*. *aureus*/MRSA. (DOCX)
