# Correction: Antimicrobial resistance profile of *Staphylococcus aureus* isolated from patients, healthcare workers, and the environment in a tertiary hospital in Addis Ababa, Ethiopia

**DOI:** 10.1371/journal.pone.0324658

**Published:** 2025-05-20

**Authors:** Rajiha Abubeker Ibrahim, Shu-Hua Wang, Wondwossen A. Gebreyes, Jose R. Mediavilla, Gadissa Bedada Hundie, Zelalem Mekuria, Rozina Ambachew, Dejenie Shiferaw Teklu, Barry Kreiswirth, Eyasu Tigabu Seyoum, Degefu Beyene, Nega Berhe

Eyasu Tigabu Seyoum is not included in the author byline. Eyasu Tigabu Seyoum should be listed as the tenth author and affiliated with Ohio State Global One Health (GOH) LLC, Addis Ababa, Ethiopia. The contributions of this author are as follows: Conceptualization.
