# Supplementary material for: Antimicrobial resistance profile of Staphylococcus aureus isolated from patients, healthcare workers, and the environment in a tertiary hospital in Addis Ababa, Ethiopia
Source: PLoS One. 2024 Aug 15;19(8):e0308615. doi: 10.1371/journal.pone.0308615 (PMC11326609; doi:10.1371/journal.pone.0308615)
Supplement: S1 Table — (DOCX) [file pone.0308615.s001.docx]

**Supplement 1. Demographics of health care workers and positive isolates from each sample and correlation of the age, sex, department, work experience, profession and level of education to isolation of *S. aureus*/MRSA.**

| Healthcare workers N=202 | | *Staphylococcus aureus* | | | MRSA | | |
| --- | --- | --- | --- | --- | --- | --- | --- |
| Sex n (%) | | **n (%)** | **OR** | **p** | **n (%)** | **OR** | **p** |
| Female | 99 (49.0) | 26 (51.0) | NA | NA | 1 (20.0) | NA | NA |
| Male | 103 (51.0) | 25 (49.0) | 0.855 | 0.626 | 4 (80.0) | 4.76 | 0.177 |
| Age n (%) | | ***S. aureus*** | **OR** | **p** | **MRSA** | **OR** | **p** |
| 21-29 | 133 (65.8) | 33 (65.0) | 3.77 | 0.211 | 3 (60.0) | NA | NA |
| 30-39 | 57 (28.2) | 17 (33.3) | 4.675 | 0.155 | 2 (40.0) | 1.333 | 0.766 |
| 40-49 | 12 (6.0) | 1 (2.0) | NA | NA | 0(0.0) | NA | NA |
| Department n (%) | | ***S. aureus*** | **OR** | **P** | **MRSA** | **OR** | **p** |
| ED | 21 (10.40) | 5 (9.8) | NA | NA | 1 (20.0) | NA | NA |
| GYN | 13 (6.44) | 2 (3.9) | 0.582 | 0.558 | 0 | NA | NA |
| LAB | 18 (8.91) | 8 (15.7) | 2.56 | 0.178 | 1 (20.0) | 0.571 | 0.718 |
| MED | 53 (26.24) | 16 (31.3) | 1.511 | 0.485 | 1 (20.0) | 0.267 | 0.385 |
| PED | 42 (20.79) | 9 (17.6) | 0.872 | 0.830 | 0 | NA | NA |
| SURG | 55 (27.23) | 11 (21.6) | 0.8 | 0.716 | 2 (40.0) | 0.889 | 0.931 |
| Work Experience n (%) | | ***S. aureus*** | **OR** | **p** | **MRSA** | **OR** | **p** |
| 0-2 | 68 (33.7) | 15 (29.4) | 1.230 | 0.857 | 3 (40.0) | NA | NA |
| 3-5 | 100 (49.5) | 27 (52.9) | 1.479 | 0.731 | 2 (40.0) | 0.52 | 0.536 |
| 6-10 | 29 (14.5) | 8 (15.7) | 1.52 | 0.724 | 1 (20.0) | 0.928 | 0.955 |
| 11-20 | 5 (2.5) | 1 (2) | NA | NA | 0 | NA | NA |
| Profession n (%) | | ***S. aureus*** | **OR** | **p** | **MRSA** | **OR** | **p** |
| Cleaner | 19 (9.4) | 5 (9.6) | 0.46 | 0.117 | 0 | NA | NA |
| Nurse | 54 (26.7) | 13 (25.5) | 0.689 | 0.523 | 1 (20) | 0.416 | 0.500 |
| Intern | 42 (20.8) | 10(19.6) | 0.677 | 0.524 | 1(20) | 0.555 | 0.653 |
| Laboratorians | 20 (9.9) | 8(15.7) | 1.44 | 0.971 | 1(20) | 0.714 | 0.799 |
| Resident doctor | 45 (22.3) | 12(23.5) | 0.787 | 0.470 | 2(40) | NA | NA |
| Specialized doctor | 22 (10.9) | 3(5.9) | 0.342 | 0.271 | 0 | NA | NA |
| Educational level n (%) | | ***S. aureus*** | **OR** | **p** | **MRSA** | **OR** | **p** |
| Diploma | 20 (9.9) | 4(7.8) | 0.5 | 0.501 | 0 | NA | NA |
| First degree | 83 (41.6) | 26(51.0) | 0.804 | 0.903 | 3(60) | 2.190 | 0.437 |
| Second degree | 51 (25.3) | 9(17.6) | 0.428 | 0.368 | 2(40) | NA | NA |
| Third degree | 6 (3.0) | 2(3.9) | NA | NA | 0 | NA | NA |
| Other | 15 (7.4) | 5(9.8) | 1.333 | 0.777 | 0 | NA | NA |
| Intern | 26 (12.9) | 5(9.8) | 0.428 | 0.458 | 0 | NA | NA |

**Notes**: 95% Confidence Interval were used. NA, not applicable; OR, odds ratio; p, p-value;

**Abbreviations:** ED, Emergency Department; GYN, Gynecology ward; MED, Medicine ward; OPD, outpatient unit; PED, Pediatric ward; SURG, Surgical ward.
